# NET-related gene signature for predicting AML prognosis

**DOI:** 10.1038/s41598-024-59464-y

**Published:** 2024-04-20

**Authors:** Jiajia Wang, Huiping Wang, Yangyang Ding, Xunyi Jiao, Jinli Zhu, Zhimin Zhai

**Affiliations:** 1 Department of Hematology, The Second Affiliated Hospital of Anhui Medical University, Hefei, 230601 Anhui China; 2 Center of Hematology Research, Anhui Medical University, Hefei, 230601 Anhui China; 3 Department of Hematology, Tongling People’s Hospital, Tongling, 244000 Anhui China

**Keywords:** Neutrophil extracellular traps, AML, Prognostic model, Tumor microenvironment, Immunotherapy, Cancer microenvironment, Cancer models, Haematological cancer, Computational biology and bioinformatics

## Abstract

Acute Myeloid Leukemia (AML) is a malignant blood cancer with a high mortality rate. Neutrophil extracellular traps (NETs) influence various tumor outcomes. However, NET-related genes (NRGs) in AML had not yet received much attention. This study focuses on the role of NRGs in AML and their interaction with the immunological microenvironment. The gene expression and clinical data of patients with AML were downloaded from the TCGA-LAML and GEO cohorts. We identified 148 NRGs through the published article. Univariate Cox regression was used to analyze the association of NRGs with overall survival (OS). The least absolute shrinkage and selection operator were utilized to assess the predictive efficacy of NRGs. Kaplan–Meier plots visualized survival estimates. ROC curves assessed the prognostic value of NRG-based features. A nomogram, integrating clinical information and prognostic scores of patients, was constructed using multivariate logistic regression and Cox proportional hazards regression models. Twenty-seven NRGs were found to significantly impact patient OS. Six NRGs—CFTR, ENO1, PARVB, DDIT4, MPO, LDLR—were notable for their strong predictive ability regarding patient survival. The ROC values for 1-, 3-, and 5-year survival rates were 0.794, 0.781, and 0.911, respectively. In the training set (TCGA-LAML), patients in the high NRG risk group showed a poorer prognosis (*p* < 0.001), which was validated in two external datasets (GSE71014 and GSE106291). The 6-NRG signature and corresponding nomograms exhibit superior predictive accuracy, offering insights for pre-immune response evaluation and guiding future immuno-oncology treatments and drug selection for AML patients.

## Introduction

Acute Myeloid Leukemia (AML) is a malignant hematopoietic tumor characterized by the accumulation of undifferentiated and functionally heterogeneous leukemia cells. The annual incidence rate is approximately 4.3 per 100,000, with a median age of around 68 years, and the incidence increases with advancing age^1^. In the UK, Canada, Australia, and Sweden, the median age for AML diagnosis is reported to be between 63 and 71 years. In contrast, the median age at AML diagnosis in India, Brazil, and Algeria was 40, 42 and 45 years, respectively^2^. Concurrently, Clinical observations indicate a trend of decreasing age at AML diagnosis in China. Following induction therapy, 81.5% of APL patients and 62.4% of non-APL patients achieved complete response (CR)^3,4^. However, a significant proportion of patients still experience refractory or relapsed disease with a poor prognosis. Thus, the development of novel markers for assessing patient prognosis is of paramount importance.

Neutrophils, the predominant immune cells in both bone marrow and peripheral blood, play multifaceted roles in the initiation, progression, and metastasis of cancer ^5^. As integral components of the inflammatory cell population within the tumor microenvironment, neutrophils exhibit dual functions, contributing to both the promotion and suppression of cancer^5,6^. Substantial clinical evidence supports the proposition that neutrophils actively facilitate cancer progression in solid tumors^7^. In our preliminary research, a distinct subset of neutrophils was identified in the peripheral blood of B-cell non-Hodgkin lymphoma patients. This subset demonstrated a notably low proportion in healthy individuals but exhibited a significant increase in B-cell non-Hodgkin lymphoma patients^8^. Moreover, its heightened presence correlated closely with disease progression and prognosis, underscoring the distinctive value of this neutrophil subset in the context of AML^9^. Neutrophils manifest their activities not only through degranulation and phagocytosis but also by generating neutrophil extracellular traps (NETs), which efficiently neutralize bacterial virulence factors and confer bactericidal effects^10,11^. Additionally, the confirmation of NETs has been established in Diffuse Large B-Cell Lymphoma (DLBCL)^12^. Consequently, we hypothesize that NETs could serve as innovative prognostic markers in AML.

In our study, 6 robust NRGs were assessed to establish a prognostic model via TCGA-LAML cohort, and we computed a risk score to systematically examine the association between NRGs and the immune microenvironment, as well as their implications in immunotherapy and chemotherapy sensitivity. Our objective is to illustrate the significance of NRGs in evaluating the prognostic outlook for AML patients by conducting a thorough analysis of genomic data. Additionally, we seek to devise novel tools to enhance treatment strategies.

## Methods

### Data collection and processing

We obtained gene expression profiles and clinical data for the TCGA-LAML cohort, comprising 136 patients from the UCSC Xena database (https://xenabrowser.net/datapages/). For enhanced study accuracy, 19 patients lacking survival data were excluded. Concurrently, we retrieve two datasets from the GEO database (https://ncbi.nlm.nih.gov/geo/), namely GSE71014 (n = 104) and GSE106291 (n = 250). These datasets encompass gene expression profiles and clinical information related to acute myeloid leukemia (AML).In our study, we selected TCGA-LAML as the training dataset and GSE71014 and GSE106291 as the external validation datasets.

### Obtaining a list of NET genes

We chose 148 genes for examination by manually gathering previously reported NRGs from the literature. Refer to Additional file 1: Table S1 for a more detailed visual representation.

### Identification of NRGs associated with overall survival (OS)

Univariate Cox regression was employed to identify NETs associated with overall survival (OS) in the TCGA-LAML cohort (n = 136; *p* < 0.1).

### Model construction and validation for patients with AML

The training group data underwent LASSO regression analysis using the “glmnet” R package, resulting in optimal outcomes. We obtained Six NRGs and correlation coefficients. Then, we calculated each patient’s risk score. The calculation formula is as follows: Risk score = (− 2.23833 × expression level of CFTR) + (0.222962 × expression level of ENO1) + (0.201441 × expression level of PARVB) + (0.069133 × expression level of DDIT4) + (-0.05161 × expression level of MPO) + (0.046466 × expression level of LDLR). Using the quartile risk score as a cutoff, patients in the training cohort were classified into high- and low-risk groups. Kaplan–Meier survival analysis was conducted, and a receiver operating characteristic curve (ROC) was generated. To validate the model’s predictive capacity, GSE71014 (n = 104) and GSE106291 (n = 250) served as independent validation sets. Individual risk scores were computed, and Kaplan–Meier survival curves were employed to illustrate their performance in overall survival (OS). The prognostic predictive capability of NRG-based features was evaluated through time-dependent receiver operating characteristic (ROC) curves.

### Independent prognostic analysis and nomogram construction

To assess the standalone predictive potential of the NRG signature in AML patients, we performed univariate and multivariate Cox regression analyses. A nomogram was constructed using the “rms” R package to predict 1-year, 3-year, and 5-year overall survival (OS) in AML patients. The variables considered for prediction included patient age, gender, race, FAB, WBC, HB, PLT and risk scores.

### Identification of differentially expressed genes (DEGs)

We used the R package "DESeq2" to detect differentially expressed genes (DEGs) between high- and low-risk groups, with DEGs defined by |log2FC|≥ 1 and *p* < 0.05.

### Functional enrichment analysis

GO and KEGG pathway analyses were conducted utilizing the “ClusterProfiler” R package. We employed GSEA to investigate the differentially enriched KEGG pathways between high- and low-risk groups.

### Risk model’s association with TME

We used the R package “IOBR” to analyze the differences in the immune microenvironment between two groups. Using the estimate method, we assessed the relationship between risk signature and TME (the stromal score, immune score, and ESTI MATE score).

### Analysis of tumor-infiltrating immune cells and immune checkpoints

Differences in immune infiltrating cells of two risk groups were analyzed and compared using ssGSEA (single sample Gene Set Enrich ment Analysis) and CIBERSORT to seek the relationships between the risk model and immune status. The levels of 28 immune checkpoint genes were also compared between the two risk groups.

### Drug predictive analysis

We applied the model directly to the cell line expression data using the Cancer Cell Line Encyclopedia (CCLE) (https://sites.broadinstitute.org/ccle). The cell lines were categorized into high- and low-risk groups, and the differences in drug sensitivity between the two groups were compared.

### Statistical analysis

All statistical analyses were performed using the R (version 4.2.2) software. *P* values < 0.05 were considered significant (*P < 0.05, **P < 0.01, ***P < 0.001).

## Results

### NET-related genes with significant prognostic value in AML

**Figure 1:**
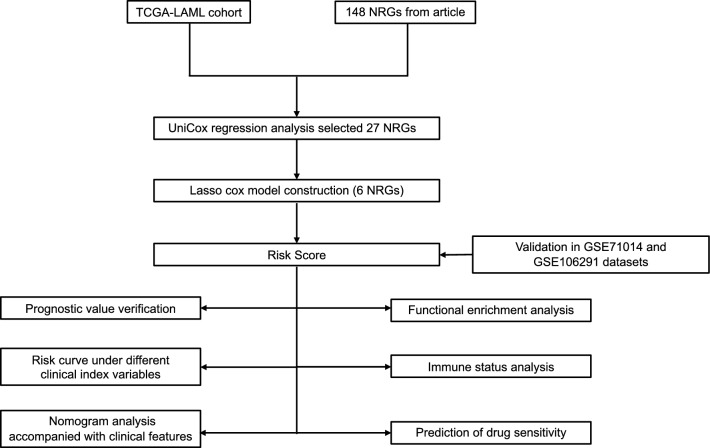
Study flow chart.

**Figure 2:**
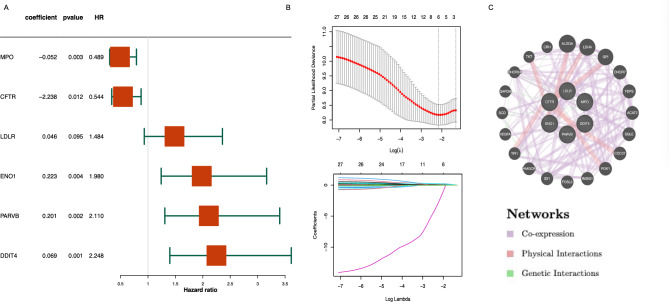
Identification of NET-related genes in AML patients. (**A**) The six genes were significantly associated with prognosis after univariate Cox and Lasso Cox analysis. The HR of LDLR, ENO1, PARVB and DDIT4 is higher than 1, and the MPO and CFTR for HR is less than 1. (**B**) Lasso Cox regression for 27 NRGs in univariate Cox regression. (**C**) The protein interaction network of the 6-NRGs.

The analysis of gene signatures related to NETs is outlined through a systematic workflow, as depicted in Fig. 1. We collated clinical and survival data for 136 patients from TCGA, as detailed in Table 1. Through literature review, we identified 148 NRGs, as shown in Table S1.Univariate Cox analysis (*P* < 0.1) validated 27 NRGs as significantly prognostic for AML patients (Table S2; Fig. S1). Utilizing Lasso Cox analysis, we pinpointed six genes with prognostic significance, illustrated in Fig. 2A,B. Those genes are CFTR, ENO1, PARVB, DDIT4, MPO, and LDLR. The protein interaction network among the genes CFTR, ENO1, PARVB, DDIT4, MPO, and LDLR is depicted in Fig. 2C. Four genes, LDLR, ENO1, PARVB, and DDIT4, were identified as adverse prognostic markers, while MPO and CFTR were found to be favorable for prognosis.

**Table 1:** Clinical pathological parameters of AML.

	Overall (n = 136)
Age	
Mean(SD)	54.60 (15.95)
Median[IQR]	56.50 [42.75, 67.00]
FAB	
M0 Undifferentiated	14 (10.3)
M1	31 (22.8)
M2	35 (25.7)
M3	12 (8.8)
M4	27 (19.9)
M5	13 (9.6)
M6	2 (1.5)
M7	1 (0.7)
Not classified	1 (0.7)
Gender	
Female	59 (43.4)
Male	77 (56.6)
Race	
Asian	1 (0.7)
Black or african american	13 (9.6)
Not reported	1 (0.7)
White	121 (89.0)
Plt	
Mean(SD)	65.65 (55.03)
Median[IQR]	45.50 [27.00, 87.00]
HB	
Mean(SD)	9.55 (1.42)
Median[IQR]	9.00 [9.00, 10.00]
WBC	
Mean(SD)	35.07 (42.42)
Median[IQR]	15.00 [4.50, 49.00]
OS.status	
Alive	45 (33.1)
Dead	72 (52.9)
Missing	19 (14.0)

### Validation of the NRGs signature

**Figure 3:**
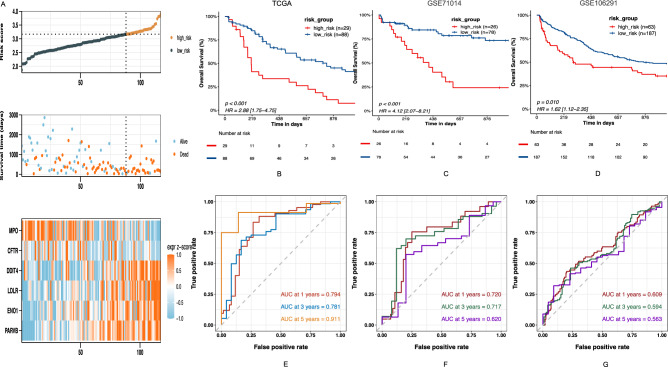
Prognosis value of the six NRGs model in the training set and validation sets. (**A**) Exhibition of predictive model based on risk score of the training set, survival time and survival status between high- and low-risk groups in the training set, The heatmap of 6-NRGs in the training set. (**B**–**D**) Kaplan–Meier survival curves of OS between high- and low-risk groups in the training set and validation sets, respectively. (**E**–**G**) Time-dependent ROC curves of 1-, 3-, and 5-years of AML patients in the training set and validation sets, respectively.

To validate the stability and generalizability of our model, we employed the TCGA-LAML cohort as the internal training set and the GSE71014 and GSE106291 cohorts for external validation. Risk scores were calculated separately for each sample in the TCGA training cohort based on the risk formula (Table S3). Elevated risk scores in the AML patient training set were associated with decreased OS and higher mortality rates. Based on the quartile risk score, Patients were categorized into high and low-risk groups to investigate prognostic differences (Fig. 3A). Kaplan–Meier analysis showed improved OS in the low-risk group compared to the high-risk group in both training and validation sets (Fig. 3B,C,D; *P* < 0.001, *P* < 0.001, *P* = 0.010, respectively). The ROC curve predicted patient survival at 1-, 3-, and 5-year intervals, with TCGA-LAML cohort AUCs of 0.794, 0.781, and 0.911, respectively (Fig. 3E). The AUCs for the GSE71014 and GSE106291 cohorts were illustrated in Fig. 3F,G. This demonstrates the model’s predictive capability.

### Construction a prognostic nomogram with clinical characteristics

**Figure 4:**
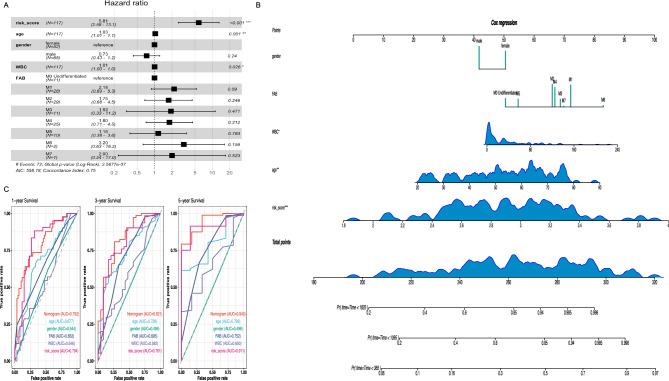
Assessment of the predictive risk model of the six NRGs in AML. (**A**) The multivariate Cox regression analysis of risk model score and clinical features regarding prognostic value. (**B**) A clinical prognostic nomogram was developed to predict 1-, 3-, and 5-year survival. (**C**) Time-dependent ROC curves for 1-, 3-, and 5-year outcomes of AML patients, based on a nomogram, risk score, and clinical information.

To confirm the reliability and clinical applicability of the NRGs for prognostic prediction, we compared AML patients' risk scores with standard clinical indicators and assessed their correlation with patient outcomes using multivariate Cox analysis. The results of the multivariate Cox analysis clearly indicate that the risk score (*P* < 0.001) is a significant prognostic factor for patient outcomes (Fig. 4A). Following this analysis, we combined the risk score with clinical indicators to create Nomogram plots (Fig. 4B; Table S4), enabling quantitative prognosis prediction and aiding clinical decision-making. These plots estimate survival probabilities at 1, 3 and 5 years. Concurrently, the nomogram’s 1-, 3-, and 5-year ROC values were 0.792, 0.821, and 0.940, respectively (Fig. 4C). The nomogram effectively predicts the survival outcomes of AML patients.

### Functional enrichment analysis of risk model

**Figure 5:**
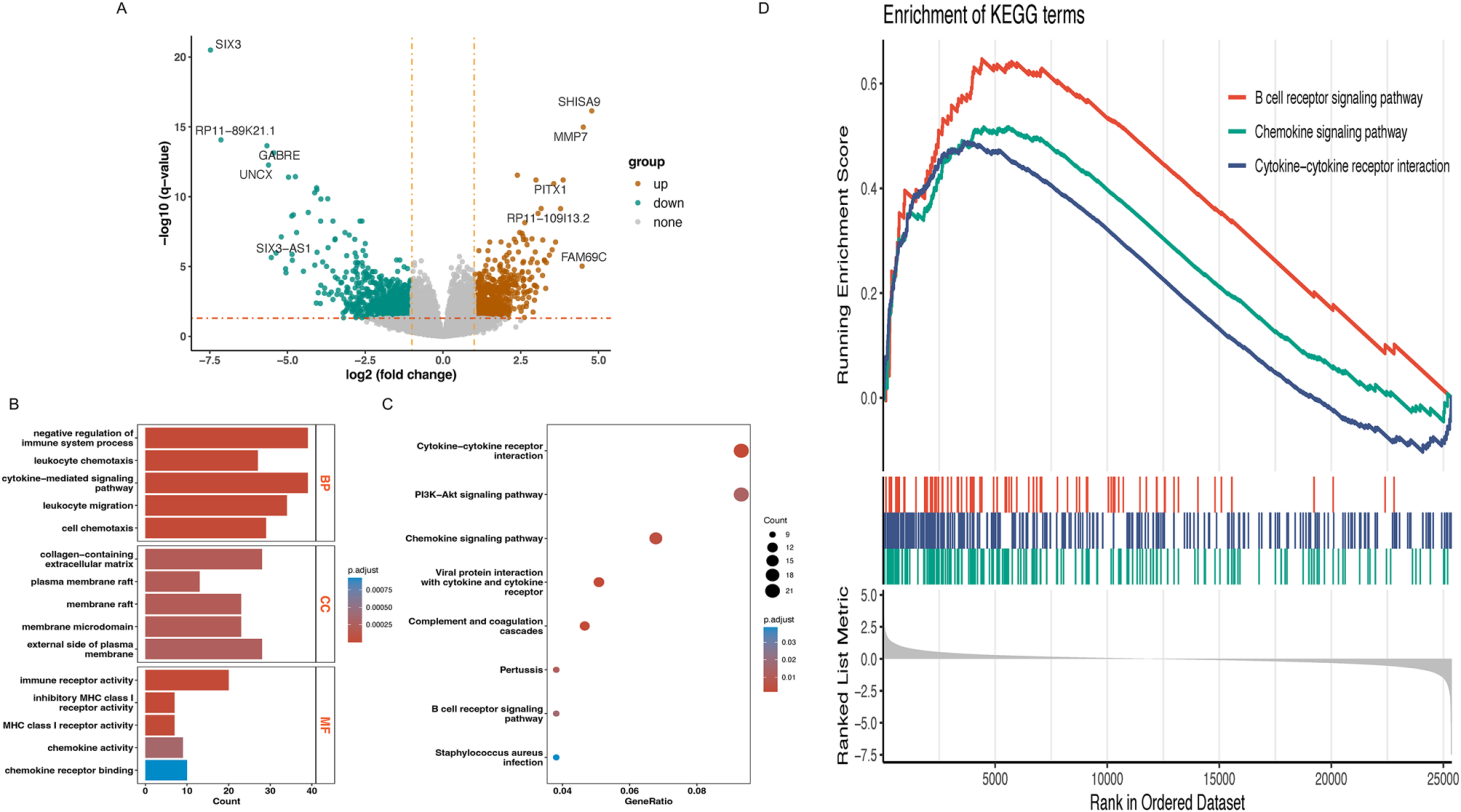
The functional enrichment analysis based on the six NRGs model. (**A**) DEGs associated with biological characteristics between high- and low-risk patients. (**B**) GO terms (**C**) KEGG pathways (**D**) GSEA analysis.

We identified DEGs associated with biological characteristics between high- and low-risk patients (Fig. 5A; Table S5). Enrichment analysis was conducted, encompassing GO terms (biological process, cellular component, molecular function) and KEGG pathways (Table S6). Identified GO terms in biological processes include those related to immune system process, leukocyte chemotaxis, cytokine—mediated signaling pathway, leukocyte migration, cell chemotaxis. Significant enrichment in the molecular function subontology was noted in immune receptor activity, inhibitory MHC class I receptor activity, MHC class I receptor activity, chemokine activity and chemokine receptor binding. Cellular component enrichment indicated DEGs involvement in structures like the collagen—containing extracellular matrix, plasma membrane raft, membrane raft, membrane microdomain and external side of plasma membrane (Fig. 5B). KEGG pathway analysis suggested DEGs involvement in pathways such as Cytokine-cytokine receptor interaction, PI3K-Akt signaling pathway, Chemokine signaling pathway, Viral protein interaction with cytokine and cytokine receptor, Complement and coagulation cascades, Pertussis, B cell receptor signaling pathway (Fig. 5C)^13–15^. For the high- and low-risk group, the differentially enriched KEGG pathways between the two groups were analyzed by GSEA.B cell receptor signaling pathway, Chemokine signaling pathway, Cytokine-cytokine receptor interaction were the pathways that were substantially enriched in the high-risk group (Fig. 5D)^13–15^.

### Immunity analysis of the risk signature

**Figure 6:**
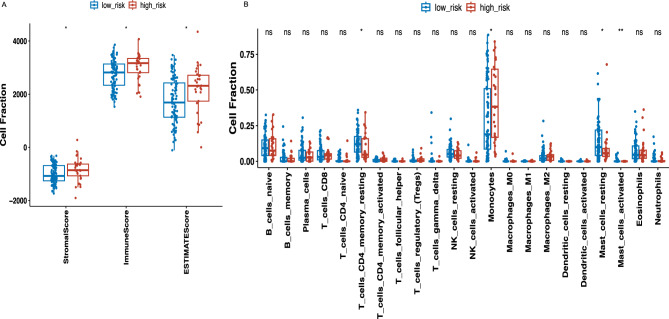
Immune cell infiltration in different risk groups. (**A**) Estimate Score, Immune Score and Stromal Score in different risk groups (**B**) The CIBERSORT algorithm determined the differences between the two groups. *P* value < 0.05 indicates statistical significance. *P < 0.05; **P < 0.01; ***P < 0.001; ns, non-significant.

**Figure 7:**
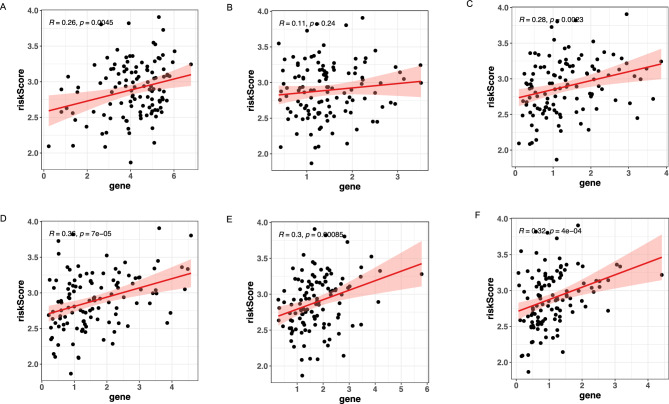
Pearson correlation of the risk scores and immune checkpoint genes. (**A**) HAVCR2 (**B**) TIGIT (**C**) CTLA4 (**D**) LAG3 (**E**) PDCD1 (**F**) CD274.

We further investigated the relationships within the Tumor Microenvironment (TME), as measured by the Estimate Score, Immune Score, and Stromal Score, between the two groups. Patients in the high-risk group had higher estimate scores, immune scores, and stromal scores, compared to patients in the low-risk group (*P* < 0.05 for all) (Fig. 6A). This outcome suggests that patients in the high-risk group may be particularly suitable candidates for specific types of immunotherapies. Next, we used CIBERSORT to explore the difference in immunity levels between the two groups. By CIBERSORT, the abundance levels of Monocytes were significantly higher in the high-risk group (*P* < 0.05). In contrast, T_cells_CD4_memory_resting, Mast_cells_resting and Mast_cells_activated were enriched in the low-risk group (all *P* < 0.05) (Fig. 6B). It shows no significant differences among neutrophils. Monocytes, mast cells, and neutrophils, all crucial white blood cell types, play key roles in the immune response^16^. Despite their differing functions and activities, these cells share numerous granule components, indicating potential immune response interactions. Typically, these granules contain enzymes, cytokines, and bioactive substances crucial for defense, inflammation, signaling, and repair. Shared granules suggest these cells have similar immune functions, like engaging in inflammation and fighting pathogens^17,18^. Thus, significant differences in monocytes and mast cells indirectly corroborate the study's findings to some extent.Due to the significant impact of abnormal expression and function of immune checkpoint molecules on tumor immunotherapy, we analyzed correlation between immune checkpoint genes and risk score (Fig. 7). The risk score was positively correlated with the immune checkpoint genes of CD274 (R = 0.32, *p* = 4e − 04), PDCD1(R = 0.3, *p* = 0.00085) and LAG3 (R = 0.36, *p* = 7e − 05). This suggests that targeted therapies against CD274, PDCD1 and LAG3 may be able to benefit patients in the high-risk group.

### Drug predictive analysis of risk model

**Figure 8:**
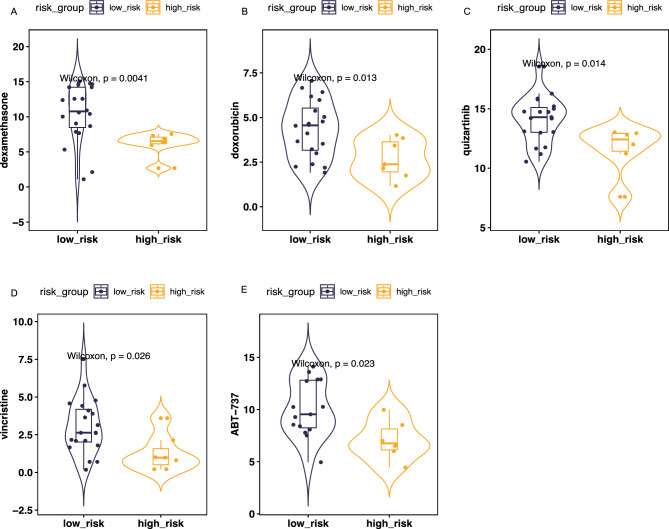
Drug predictive analysis by CCLE database. (**A**) dexamethasone (**B**) doxorubicin (**C**) quizartinib (**D**) vincristine (**E**) ABT-737. *P* value < 0.05 indicates statistical significance.

Treatment approaches for high-risk patients were explored using CCLE database acute myeloid leukemia cell line data. After classifying the cell lines into high and low-risk categories based on risk scores, we predicted the outcomes of drug treatments. Our findings indicate that dexamethasone (*P* = 0.0041), doxorubicin (*P* = 0.013), quizartinib (*P* = 0.014), vincristine (*P* = 0.026) and ABT-737 (*P* = 0.023) exhibit enhanced prognostic effectiveness in high-risk groups (Fig. 8).

## Discussion

AML comprises a diverse group of primary hematopoietic neoplasms originating from myeloid precursor cells. Approximately 50% of patients do not achieve remission with initial therapy, subsequently developing refractory AML^19,20^. In our study, we specifically investigated the impact of NRGs on the prognosis of AML patients. We developed a risk prediction model utilizing six OS-related NRGs, which demonstrated independent prognostic value in comparison to clinical features. In summary, our research has identified a novel, reliable biomarker for prognostic assessment in AML patients.

We constructed a six gene risk model in the training set through univariate and LASSO Cox regression analyses. The obtained risk score functioned as an independent prognostic marker. ROC curve analysis demonstrated the model's significantly superior prognostic performance. We developed a nomogram that includes patients' clinical information and prognostic scores, enhancing the model's clinical utility. Model validation in independent sets confirmed its excellent predictive performance. To our knowledge, this study is the first to evaluate the role of NRGs in AML. The NETs risk model remains highly relevant in Diffuse Large B-cell Lymphoma and other solid tumors. Shi et al. developed a prognostic model based on NETs for DLBCL, achieving AUCs of 0.80, 0.82, and 0.79 at 1, 3, and 5 years in the training set, respectively^12^. Similarly, Xin et al. developed a prognostic model based on NETs for hepatocellular carcinoma, with AUCs of 0.836, 0.879, and 0.902 at 1, 3, and 5 years in the training set, respectively^21^. Zhao et al.’s NRGs risk model in breast cancer demonstrated good predictive performance, with respective AUCs of 0.73, 0.80, and 0.78^22^. These studies suggest that prognostic models based on NETs may hold potential prognostic significance in hematologic malignancies and solid tumors.

In the last 20 years, there has been a significant increase in interest to define the role of neutrophils more clearly in modulating immune responses^23^. The Granulocytic Myeloid-Derived Suppressor Cells (G-MDSCs) are of primary importance in these studies^24^. This subgroup of Myeloid-Derived Suppressor Cells predominantly consists of granulocytic cells, including neutrophils^25,26^. G-MDSCs primarily function to suppress the host’s immune response using diverse mechanisms, which aids in tumor growth and metastasis^25^. Furthermore, G-MDSCs promote tumor angiogenesis and tissue remodeling, creating an environment conducive to tumor growth and metastasis^24,25^. In our preliminary research, we observed that patients with G-MDSCs (%) ≥ 98.70% in the newly diagnosed B-NHL subgroup had a shorter overall survival time compared to those with G-MDSCs (%) < 98.70%. Additionally, a notable survival difference was found in patients with M-MDSCs (%) ≥ 7.19% versus those with M-MDSCs (%) < 7.19% in the relapsed B-NHL subgroup^8^. Recent studies have demonstrated that mature CD10 + and immature CD10- neutrophils in G-CSF–treated donors exhibit contrasting impacts on T cells^9^. Consequently, genes associated with neutrophils hold significant potential as effective markers for assessing tumor patients.

NETs, composed of chromatin and antimicrobial proteins, are released by activated neutrophils. Recent evidence demonstrates NETs’ role in cancer progression and metastasis in both animal models and patients^27^. This study suggests that six NRGs—CFTR, ENO1, PARVB, DDIT4, MPO, LDLR—could influence NET formation, building on previous research. CFTR-expressing innate immune cells show increased neutrophil recruitment and enhanced pro-inflammatory cytokine production in response to inflammatory challenges^28^. ENO1, a glycolysis enzyme, also plays extracellular roles in extracellular matrix assembly and immune regulation^29^. As a cytoskeletal component, PARVB contributes to cell adhesion and migration, which may indirectly influence NET formation due to the cytoskeleton’s role in NET release^30^. DDIT4, which regulates the mTOR signaling pathway in response to stress and DNA damage, may indirectly influence NET release by affecting neutrophil stress responses and survival^31^. MPO, a key lysosomal enzyme in neutrophils, is essential for NET formation and stabilizes NET structures by producing antimicrobial agents^32^. While LDLR's direct role in NET formation is unexplored, its involvement in cholesterol metabolism and intracellular signaling could indirectly influence neutrophil functionality and NET release^33^.

Genes within the NRG risk model exhibit a range of functions during disease. PARVB plays a role in actin reorganization and focal adhesion, contributing to cell adhesion, spreading, and motility^30^. Studies indicate that overexpression of PARVB can facilitate the endogenous growth and metastasis of tongue squamous cell carcinoma through enhanced tumor migration^34^. In urothelial cancer, PARVB downregulation is linked to increased cell proliferation and migration^35^. This study associates PARVB with a poor prognosis in AML, but further research is needed to elucidate its exact mechanism. Researchers have identified CFTR as a tumor suppressor gene in both murine and human^36^. CFTR, a glycoprotein with 1480 amino acids, belongs to the ATP-binding cassette (ABC) transporter superfamily and functions as a cAMP-dependent Cl- channel, mediating the transport of Cl- and HCO3- intestinal cancer, and studies suggest that CFTR-deficient tumors may be driven by the activation of β-catenin^36–38^. Similarly, our research indicates that CFTR is associated with a favorable prognosis in AML. ENO1 (2-phospho-D-glycerate hydrolase) is an enzyme in glycolysis, catalyzing the conversion of 2-phosphoglyceric acid to phosphoenolpyruvic acid^29^. ENO1 enhances tumor-related cellular activities such as increased glycolysis, cancer cell proliferation, migration, invasion, drug resistance, and oncogenic signaling pathway activation^29,39^. Additionally, ENO1's cell surface localization renders it a promising prognostic and diagnostic biomarker for cancer^40^. DNA damage inducible transcript 4 (DDIT4), an inhibitor of the mammalian target of rapamycin (mTOR), is expressed in response to various cellular stresses^41^. Research indicates that in various malignancies, DDIT4 is involved in tumorigenesis and influences patient survival^42–44^. Studies have confirmed that high DDIT4 expression may be a poor prognostic indicator for AML^45^. MPO, a lysosomal enzyme produced by myeloid cells, is primarily found in neutrophils and monocytes^46^. It plays a crucial role in anti-infection immune responses and serves as a key marker of myeloid cell differentiation^46,47^. MPO is a vital differentiation marker in diagnosing AML, with its expression levels typically elevated, particularly in AML-M3^46,48^. LDLR, a cell-surface glycoprotein, facilitates the endocytosis of cholesterol-rich low-density lipoprotein (LDL)^33^. In some solid tumors, elevated LDLR expression and LDL uptake have been associated with tumor progression in vivo^49,50^. It has been proposed that LDL uptake by AML cell lines may contribute to chemotherapy resistance in vitro. LDLR is an independent adverse prognostic factor in AML^51^. In conclusion, while some genes' roles in AML remain unexplored, our study identified a potential link between six prognostic genes and AML prognosis, potentially guiding future research.

Chemotherapy remains the first-line treatment of choice for AML patients, yet a significant number of patients experience relapse post-treatment. Recent research has validated the safety and effectiveness of immune checkpoint inhibitors in AML patients, indicating their potential as adjunctive therapies^52,53^. Given the positive correlation between CD274, PDCD1, and LAG3 genes and the risk score, inhibiting these genes may benefit patients.

Additionally, for high-risk groups identified by the predictive model, we utilized the CELL database to identify beneficial treatments. Dexamethasone, doxorubicin, quizartinib, vincristine and ABT-737 were found to be advantageous for high-risk groups within the model. Dexamethasone, a widely used synthetic glucocorticoid, exhibits potent anti-inflammatory and immunosuppressive effects^54^. Studies have demonstrated that dexamethasone enhances the anti-tumor efficacy of the BCL-2 inhibitor venetoclax. Dexamethasone alone has minimal impact on AML cell viability, but in combination with venetoclax, it significantly increases venetoclax-induced apoptosis in AML cells^55^. The combined use of dexamethasone and immune inhibitors undoubtedly presents promising research opportunities. The mechanism of action of doxorubicin primarily involves intercalating into the DNA double helix and inhibiting topoisomerase II activity, thereby blocking DNA replication and transcription, and consequently inhibiting tumor cell proliferation and inducing apoptosis^56^. Quizartinib’s primary mechanism involves the inhibition of FLT3 tyrosine kinase activity, thus blocking FLT3 mutation-induced signaling pathways that are essential for leukemia cell proliferation and survival^57^. Consequently, quizartinib can inhibit the growth of leukemia cells and induce apoptosis. Recent long-term clinical trials have shown that adding quizartinib to standard chemotherapy, with or without allo-HCT, and continuing monotherapy for up to three years, improves overall survival in adults aged 18–75 with newly diagnosed FLT3-ITD-positive AML^57–59^. Vincristine has been widely used for a long time in clinical treatment of malignant hematological tumors, including leukemia. It is a plant-derived alkaloid, specifically a Vinca alkaloid. Vincristine's mechanism of action hinders cancer cell division and proliferation. It disrupts the normal function of microtubule proteins, essential for cell division. Vincristine is frequently used in combination with other chemotherapeutic agents to enhance therapeutic efficacy^60^. ABT-737 is an experimental anti-cancer drug, a small-molecule inhibitor of the BCL-2 protein family^61^. The BCL-2 protein family regulates programmed cell death, and its aberrant expression is associated with tumor cell survival and drug resistance in various cancers^62^. ABT-737's mechanism of action involves mimicking the function of BH3-only proteins, which promote apoptosis. It specifically targets BCL-2, BCL-xL, and BCL-w proteins, releasing pro-apoptotic proteins, thus inducing programmed cell death in cancer cells^63^. Recently, Venetoclax, a BCL-2 inhibitor, has been utilized in clinical for treating AML, targeting BCL-2 proteins to induce apoptosis in cancer cells. As previously mentioned, Dexamethasone can enhance the anti-tumor effects of BCL-2 inhibitors^55^. Additionally, BCL-xL and BCL-w are anti-apoptotic proteins. Given the success of Venetoclax, it is anticipated that drugs targeting these proteins will be developed in the future, benefiting more patients. This provides insight into the potential effectiveness of drugs as standard treatment for AML.

However, our study had several limitations. First, the TCGA database offers limited clinical feature information and may lack other clinical parameters. Second, data from retrospective studies might be subject to selection bias. Finally, Validation assays for the gene panel within the AML risk model remain to be performed. Our research team intends to employ quantitative Reverse transcription PCR (RT-PCR) and Western blotting techniques for subsequent validation.

In conclusion, we identified NRGs linked to prognosis and developed a six-gene prognostic model. This model generates a prognostic score independent of other factors. Our study analyzed the risk model's predictive performance and screened potential treatment drugs.

### Supplementary Information

Supplementary Figure S1.

Supplementary Table S1.

Supplementary Table S2.

Supplementary Table S3.

Supplementary Table S4.

Supplementary Table S5.

Supplementary Table S6.

Supplementary Table S7.

## Data Availability

All data are available from the TCGA database (https://xenabrowser.net/datapages/) and GEO (https://ncbi.nlm.nih.gov/geo/) database within the article. GEO database under accession number GSE71014 and GSE106291.
