# Supplementary figures and images for: NET-related gene signature for predicting AML prognosis
Source: Sci Rep. 2024 Apr 20;14:9115. doi: 10.1038/s41598-024-59464-y (PMC11032381; doi:10.1038/s41598-024-59464-y)

Figure S1:27 NRGs as significantly prognostic for AML patients after univariate Cox regression

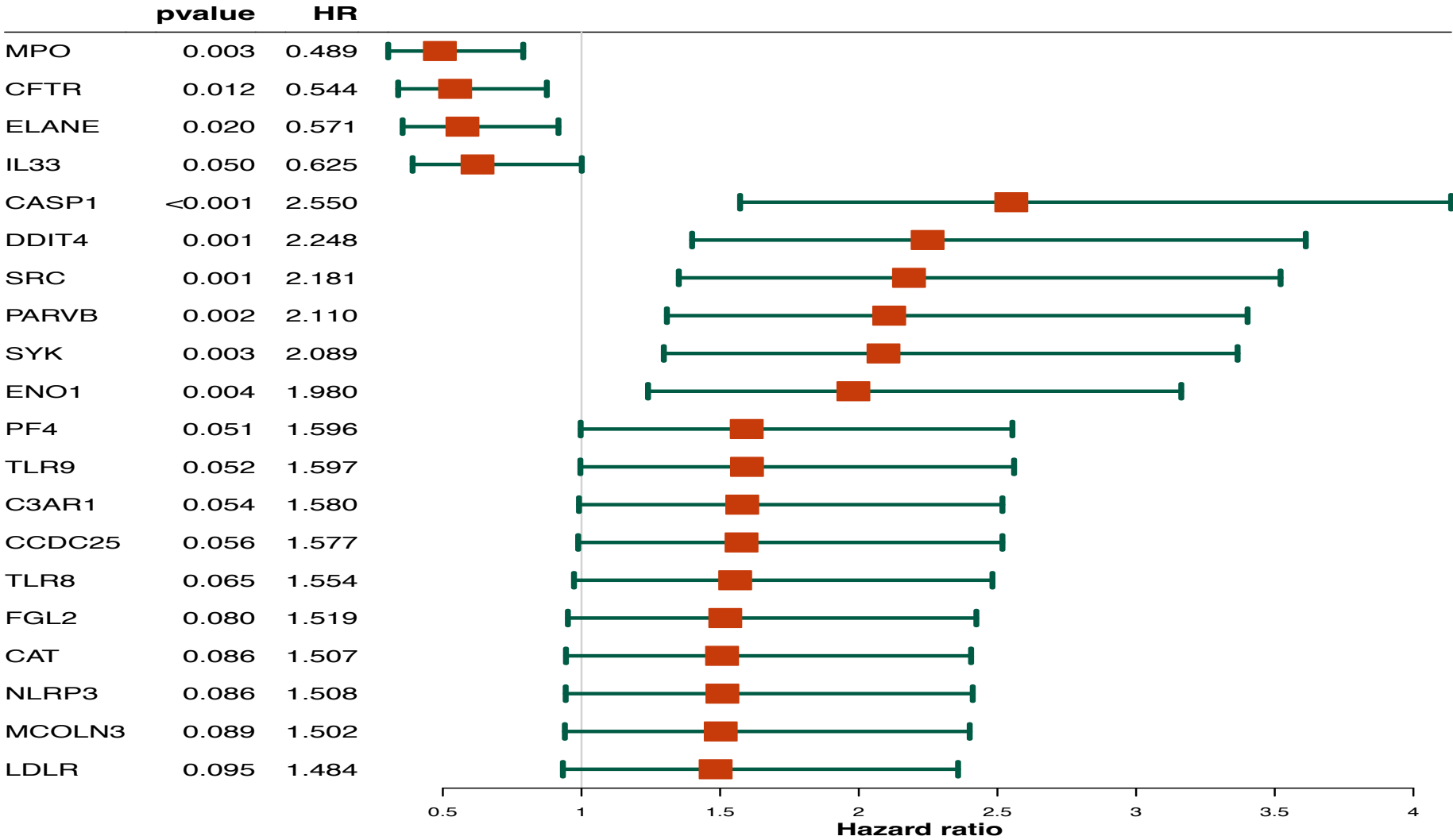

Supplement: Supplementary file 1 — Supplementary Figure S1. [file 41598_2024_59464_MOESM1_ESM.pdf]
