# Supplementary material for: NET-related gene signature for predicting AML prognosis
Source: Sci Rep. 2024 Apr 20;14:9115. doi: 10.1038/s41598-024-59464-y (PMC11032381; doi:10.1038/s41598-024-59464-y)
Supplement: Supplementary file 2 — Supplementary Table S1. [file 41598_2024_59464_MOESM2_ESM.docx]

**Table S1: Information of 148 NET-related genes (NRGs) collected in this research.**

| **Gene symbol** | **Entrez Gene** | **Description** | **Ensembl** | **Title** |
| --- | --- | --- | --- | --- |
| ACTB | 60 | Actin beta | ENSG00000075624 | Formation of neutrophil extracellular traps requires actin cytoskeleton rearrangements |
| AKT1 | 207 | AKT serine/threonine kinase 1 | ENSG00000142208 | Effects and Action Mechanism of Huoxue Tongluo Formula on the Formation of Neutrophil Extracellular Traps |
| AKT2 | 208 | AKT serine/threonine kinase 2 | ENSG00000105221 | The genomic landscape of small intestine neuroendocrine tumors |
| Ang II | 186 | Angiotensin II receptor type 2 | [ENSG00000180772](http://www.ensembl.org/id/ENSG00000180772) | Angiotensin II triggers release of neutrophil extracellular traps, linking thromboinflammation with essential hypertension |
| ANXA1 | 301 | Annexin A1 | ENSG00000135046 | Annexin A1 and Autoimmunity: From Basic Science to Clinical Applications |
| ANXA2 | 302 | Annexin A2 | [ENSG00000182718](http://www.ensembl.org/id/ENSG00000182718) | Caught in a Trap? Proteomic Analysis of Neutrophil  Extracellular Traps in Rheumatoid Arthritis and Systemic Lupus Erythematosus |
| ARPIN | 348110 | Actin related protein 2/3 complex inhibitor | ENSG00000242498 | Actin-related protein 2/3 complex regulates neutrophil extracellular trap expulsion and lung damage in abdominal sepsis |
| ATG7 | 10533 | Autophagy related 7 | ENSG00000197548 | Overexpression of ATG5 Gene Makes Granulocyte- Like HL-60 Susceptible to Release Reactive Oxygen Species |
| AZU1 | 566 | Azurocidin 1 | ENSG00000172232 | Caught in a Trap? Proteomic Analysis of Neutrophil Extracellular Traps in Rheumatoid Arthritis and Systemic Lupus Erythematosus |
| C1QA | 712 | Complement C1q A chain | ENSG00000173372 | Complement, interferon and lupus |
| C3 | 718 | Complement C3 | ENSG00000125730 | Complement and tissue factor–enriched neutrophil extracellular traps are key drivers in COVID-19 immunothrombosis |
| C3AR1 | 719 | Complement C3a receptor 1 | ENSG00000171860 | NLRP6 Serves as a Negative Regulator of Neutrophil Recruitment and Function During Streptococcus pneumoniae Infection |
| C5AR1 | 728 | Complement C5a receptor 1 | ENSG00000197405 | Complement C5a induces the formation of neutrophil extracellular traps by myeloid-derived suppressor cells to promote metastasis |
| CAMP | 820 | Cathelicidin antimicrobial peptide | ENSG00000164047 | Therapeutic Potential of Cathelicidin Peptide LL-37, an Antimicrobial Agent, in a Murine Sepsis Model |
| CAMP | 820 | Cathelicidin antimicrobial peptide | ENSG00000164047 | Neutrophil extracellular trap-associated RNA and LL37 enable self-amplifying inflammation in psoriasis |
| CARD11 | 84433 | Caspase recruitment domain family member 11 | ENSG00000198286 | The genomic landscape of small intestine neuroendocrine tumors |
| CASP1 | 834 | Caspase 1 | ENSG00000137752 | Neutrophil extracellular traps promote macrophage pyroptosis in sepsis |
| CAT | 847 | Catalase | ENSG00000121691 | Neutrophil oxidative stress mediates obesity- associated vascular dysfunction and metastatic transmigration |
| CAT | 847 | Catalase | [ENSG00000121691](http://www.ensembl.org/id/ENSG00000121691) | Neutrophil extracellular traps (NETs) modulate inflammatory profile in obese humans and mice: adipose tissue role on NETs levels |
| CCDC25 | 55246 | Coiled-coil domain containing 25 | ENSG00000147419 | DNA of neutrophil extracellular traps promotes cancer metastasis via CCDC25 |
| CCL2 | 6347 | C-C motif chemokine ligand 2 | ENSG00000108691 | Myeloid-Specific Deletion of Peptidylarginine Deiminase 4 Mitigates Atherosclerosis |
| CCL3 | 6348 | C-C motif chemokine ligand 3 | ENSG00000277632 | Immune mechanism of low bone mineral density caused by ankylosing spondylitis based on bioinformatics and machine learning |
| CCL4 | 6351 | C-C motif chemokine ligand 4 | ENSG00000275302 | Breast Cancer Cell-Neutrophil Interactions Enhance Neutrophil Survival and Pro-Tumorigenic Activities |
| CCL5 | 6352 | C-C motif chemokine ligand 5 | ENSG00000271503 | Depiction of the genomic and genetic landscape identifies CCL5 as a protective factor in colorectal neuroendocrine carcinoma |
| CD177 | 57126 | CD177 molecule | ENSG00000204936 | CD177+ cells produce neutrophil extracellular traps that promote biliary atresia |
| CD40L | 959 | CD40 ligand | ENSG00000102245 | B cell-helper neutrophils stimulate the diversification and production of immunoglobulin in the marginal zone of the spleen |
| CD44 | 960 | CD44 molecule (Indian blood group) | ENSG00000026508 | CD44/ERM/F-actin complex mediates targeted nuclear degranulation and excessive neutrophil extracellular trap formation during sepsis |
| CFB | 629 | Complement factor B | [ENSG00000243649](http://www.ensembl.org/id/ENSG00000243649) | NETosing Neutrophils Activate Complement Both on Their Own NETs and Bacteria via Alternative and Non-alternative Pathways |
| CFP | 5199 | Complement factor properdin | ENSG00000126759 | NETosing Neutrophils Activate Complement Both on Their Own NETs and Bacteria via Alternative and Non-alternative Pathways |
| CFTR | 1080 | ATP-binding cassette sub-family C, member 7 | ENSG00000001626 | Neutrophil extracellular traps and the dysfunctional innate immune response of cystic fibrosis lung disease: a review |
| CLEC4E | 26253 | Macrophage-inducible C-type lectin | ENSG00000166523 | Protective role of Mincle in bacterial pneumonia by regulation of neutrophil mediated phagocytosis and extracellular trap formation |
| CLEC7A | 64581 | Dectin-1 | ENSG00000172243 | Neutrophils sense microbe size and selectively release neutrophil extracellular traps in response to large pathogens |
| CTSC | 1075 | Cathepsin C | ENSG00000109861 | Cathepsin C promotes breast cancer lung metastasis by modulating neutrophil infiltration and neutrophil extracellular trap formation |
| CTSG | 1511 | Cathepsin G | ENSG00000100448 | Neutrophil extracellular traps can serve as platforms for processing and activation of IL-1 family cytokines |
| CXCL1 | 2919 | C-X-C motif chemokine ligand 1 | ENSG00000163739 | Myeloid-Specific Deletion of Peptidylarginine Deiminase 4 Mitigates Atherosclerosis |
| CXCL2 | 2920 | C-X-C motif chemokine ligand 2 | ENSG00000081041 | Myeloid-Specific Deletion of Peptidylarginine Deiminase 4 Mitigates Atherosclerosis |
| CXCR4 | 7852 | C-X-C motif chemokine receptor 4 | ENSG00000121966 | CXCR4 and CXCR7 Inhibition Ameliorates the Formation of Platelet-Neutrophil Complexes and Neutrophil Extracellular Traps through Adora2b Signaling |
| CXCR7 | 57007 | Atypical chemokine receptor 3 | ENSG00000144476 | CXCR4 and CXCR7 Inhibition Ameliorates the Formation of Platelet-Neutrophil Complexes and Neutrophil Extracellular Traps through Adora2b Signaling |
| CYBB | 1536 | NADPH oxidase | ENSG00000165168 | Neutrophil Extracellular Traps Activate Proinflammatory Functions of Human Neutrophils |
| DDIT4 | 54541 | Corresponding to the human genes | ENSG00000168209 | NFIL3 Facilitates Neutrophil Autophagy, Neutrophil Extracellular Trap Formation and Inflammation During Gout via REDD1-Dependent mTOR Inactivation |
| DEFA3 | 1668 | Defensin alpha 3 | ENSG00000239839 | RNA Sequencing in COVID-19 patients identifies neutrophil activation biomarkers as a promising diagnostic platform for infections |
| DNASE1 | 1773 | Deoxyribonuclease I | ENSG00000213938 | Neutrophil extracellular traps promote fibrous vascular occlusions in chronic thrombosis |
| DRP-1 | 10059 | Dynamin 1 like | [ENSG00000087470](http://www.ensembl.org/id/ENSG00000087470) | Aberrant Drp1-mediated mitochondrial division presents in humans with variable outcomes |
| ELANE | 1991 | Neutrophil elastase | ENSG00000197561 | Murine lupus is neutrophil elastase-independent in the MRL.Faslpr model |
| ENO1 | 2023 | Enolase 1 | ENSG00000074800 | Neutrophil Extracellular Traps in the Autoimmunity Context |
| ENTPD4 | 14573 | Ectonucleoside Triphosphate Diphosphohydrolase 4 | ENSG00000197217 | The critical protein interactions and structures that elicit growth deregulation in cancer and viral replication |
| ERK5 | 778597 | Mitogen-activated protein kinase | [ENSCING00000009852](http://www.ensembl.org/id/ENSCING00000009852) | Platelet TLR4-ERK5 Axis Facilitates NET-Mediated Capturing of Circulating Tumor Cells and Distant Metastasis after Surgical Stress |
| F2RL2 | 2151 | Proteinase-activated receptor-3 | ENSG00000164220 | Activated protein C inhibits neutrophil extracellular trap formation in vitro and activation in vivo |
| FCGR2B | 2213 | Fc gamma receptor IIb | ENSG00000072694 | Acute Kidney Injury Induced Lupus Exacerbation Through the Enhanced Neutrophil Extracellular Traps (and Apoptosis) in Fcgr2b Deficient Lupus Mice With Renal Ischemia Reperfusion Injury |
| FGL2 | 10875 | Fibrinogen like 2 | ENSG00000127951 | FGL2-MCOLN3-Autophagy Axis-Triggered Neutrophil Extracellular Traps Exacerbate Liver Injury in Fulminant Viral Hepatitis |
| GSDMD | 79792 | Gasdermin D | ENSG00000104518 | GSDMD-dependent neutrophil extracellular traps promote macrophage-to-myofibroblast transition and renal fibrosis in obstructive nephropathy |
| H2AX | 3014 | H2A histone family, member X | ENSG00000188486 | In Vivo Imaging of Inflamed Glomeruli Reveals Dynamics of Neutrophil Extracellular Trap Formation in Glomerular Capillaries |
| H3C14 | 126961 | H3 clustered histone 14 | [ENSG00000203811](http://www.ensembl.org/id/ENSG00000203811) | The vitals of NETs |
| HIF1A | 3091 | Hypoxia inducible factor 1 subunit alpha | ENSG00000100644 | Itaconate Suppresses Formation of Neutrophil Extracellular Traps (NETs): Involvement of Hypoxia- Inducible Factor 1α (Hif-1α) and Heme Oxygenase (HO-1) |
| HMGB1 | 3146 | High mobility group box 1 | ENSG00000189403 | Complement C5a induces the formation of neutrophil extracellular traps by myeloid-derived suppressor cells to promote metastasis |
| HRG | 3273 | Histidine rich glycoprotein | ENSG00000113905 | Histidine-Rich Glycoprotein Alleviates Liver Ischemia/Reperfusion Injury in Mice With Nonalcoholic Steatohepatitis |
| HSP90 | 179971 | Heat shock protein 90 | ENSG00000080824 | Neutrophil extracellular traps (NETs) modulate inflammatory profile in obese humans and mice: adipose tissue role on NETs levels |
| HSPE1 | 3336 | heat shock protein family E (Hsp10) member 1 | [ENSG00000115541](http://www.ensembl.org/id/ENSG00000115541) | Neutrophil extracellular traps (NETs) modulate  inflammatory profile in obese humans and mice: adipose tissue role on NETs levels |
| IL17A | 3605 | Interleukin 17 | ENSG00000112115 | REDD1/autophagy pathway promotes thromboinflammation and fibrosis in human systemic lupus erythematosus (SLE) through NETs decorated with tissue factor (TF) and interleukin-17A (IL-17A) |
| IL1RL1 | 9173 | Interleukin 1 receptor like 1 | ENSG00000115602 | Oxidized LDL induced extracellular trap formation in human neutrophils via TLR-PKC-IRAK-MAPK and NADPH-oxidase activation |
| IL-21 | 59067 | Interleukin 21 | ENSG00000138684 | B cell-helper neutrophils stimulate the diversification and production of immunoglobulin in the marginal zone of the spleen |
| IL33 | 90865 | Interleukin 33 | ENSG00000137033 | Interleukin-33 facilitates cutaneous defense against Staphylococcus aureus by promoting the development of neutrophil extracellular trap |
| IL36RN | 26525 | Interleukin 36 receptor antagonist | ENSG00000136695 | Neutrophil Extracellular Traps Promote Inflammatory Responses in Psoriasis via Activating Epidermal TLR4/IL-36R Crosstalk |
| IL5 | 3567 | Interleukin 5 | ENSG00000113525 | IL-5 enhances the resistance of Actinobacillus pleuropneumoniae infection in mice through maintaining appropriate levels of lung M2, PMN-II and highly effective neutrophil extracellular traps |
| IL8 | 3576 | Interleukin 8 | ENSG00000169429 | Neutrophil extracellular traps (NETs) modulate inflammatory profile in obese humans and mice: adipose tissue role on NETs levels |
| ILK | 3611 | Integrin linked kinase | ENSG00000166333 | DNA of neutrophil extracellular traps promotes cancer metastasis via CCDC25 |
| IRAK4 | 51135 | Interleukin 1 receptor associated kinase 4 | ENSG00000198001 | Oxidized LDL induced extracellular trap formation in human neutrophils via TLR-PKC-IRAK-MAPK and NADPH-oxidase activation |
| IRAK4 | 51135 | Interleukin 1 receptor associated kinase 4 | ENSG00000198001 | Neutrophil Extracellular Traps Promote Angiogenesis Evidence From Vascular Pathology in Pulmonary Hypertension |
| IRF1 | 3659 | Interferon regulatory factor 1 | ENSG00000125347 | IRF-1 Intervention in the Classical ROS-Dependent Release of NETs during LPS-Induced Acute Lung Injury in Mice |
| KDM6A | 7403 | lysine demethylase 6A | ENSG00000147050 | KDM6A Loss Recruits Tumor-Associated Neutrophils and Promotes Neutrophil Extracellular Trap Formation in Pancreatic Cancer |
| KLF2 | 10365 | KLF transcription factor 2 | ENSG00000127528 | KLF2 regulates neutrophil activation and thrombosis in cardiac hypertrophy and heart failure progression |
| LACTOFERRIN | 4057 | Lactotransferrin | [ENSG00000012223](http://www.ensembl.org/id/ENSG00000012223) | Lactoferrin Suppresses Neutrophil Extracellular Traps Release in Inflammation |
| LAMP-2 | 3920 | lysosomal associated membrane protein 2 | ENSG00000005893 | Neutrophil extracellular trap formation is associated with autophagy-related signaling in ANCA- associated vasculitis |
| LDLR | 3949 | Low density lipoprotein receptor | ENSG00000130164 | Cholesterol-Induced M4-Like Macrophages Recruit Neutrophils and Induce NETosis |
| LPAR3 | 23566 | Lysophosphatidic acid receptor 3 | ENSG00000171517 | Lysophosphatidic Acid Receptor 3 Suppress Neutrophil Extracellular Traps Production and Thrombosis During Sepsis |
| LYZ | 4069 | Lysozyme | ENSG00000090382 | Human peptidoglycan recognition protein S is aneffector of neutrophil-mediated innate immunity |
| MAPK1 | 5594 | Mitogen-activated protein kinase 1 | ENSG00000100030 | Streptococcus Suis Serotype 2 Stimulates Neutrophil  Extracellular Traps Formation via Activation of p38 MAPK and ERK1/2 |
| MAPK14 | 1432 | mitogen-activated protein kinase 14 | [ENSG00000112062](http://www.ensembl.org/id/ENSG00000112062) | Cineole alleviates the BPA-inhibited NETs formation by regulating the p38 pathway-mediated programmed cell death |
| MAPK14 | 1432 | Mitogen-activated protein kinase 14 | ENSG00000112062 | Triclocarban evoked neutrophil extracellular trap formation in common carp (Cyprinus carpio L.) by modulating SIRT3-mediated ROS crosstalk with ERK1/2/p38 signaling |
| MAPK3 | 5595 | Mitogen-activated protein kinase 3 | ENSG00000102882 | Neutrophil Extracellular Traps Activate Proinflammatory Functions of Human Neutrophils |
| MCOLN3 | 55283 | Mucolipin TRP cation channel 3 | ENSG00000055732 | FGL2-MCOLN3-Autophagy Axis-Triggered Neutrophil Extracellular Traps Exacerbate Liver Injury in Fulminant Viral Hepatitis |
| MFN-2 | 9927 | Mitofusin 2 | ENSG00000116688 | Neutrophil Extracellular Traps Drive Mitochondrial Homeostasis in Tumors to Augment Growth |
| MIR223 | 407008 | MicroRNA 223 | ENSG00000284567 | Neutrophil Extracellular Traps Mediate Acute Liver Failure in Regulation of miR-223/Neutrophil Elastase Signaling in Mice |
| MIR503HG | 84848 | MIR503 host gene | ENSG00000226856 | Neutrophil Extracellular Traps (NETs) Promote Non- Small Cell Lung Cancer Metastasis by Suppressing lncRNA MIR503HG to Activate the NF-κB/NLRP3 Inflammasome Pathway |
| MMP9 | 4218 | Matrix metallopeptidase 9 | ENSG00000100985 | Neutrophil extracellular traps produced during  inflammation awaken dormant cancer cells in mice |
| MNDA | 4332 | Myeloid cell nuclear differentiation antigen | ENSG00000163563 | Neutrophil proteases degrade autoepitopes of NET- associated proteins |
| MPO | 4353 | Myeloperoxidase | ENSG00000005381 | Complement C5a induces the formation of neutrophil extracellular traps by myeloid-derived suppressor cells to promote metastasis |
| MPO | 4353 | Myeloperoxidase | ENSG00000005381 | Neutrophil extracellular traps induced by IL-8 aggravate atherosclerosis via activation NF-κB signaling in macrophages |
| mtDNA | 56652 | Twinkle mtDNA helicase | [ENSG00000107815](http://www.ensembl.org/id/ENSG00000107815) | Increase of Neutrophil Extracellular Traps, Mitochondrial DNA and Nuclear DNA in Newly Diagnosed Type 1 Diabetes Children but Not in High-Risk Children |
| MTOR | 2475 | Mechanistic target of rapamycin kinase | ENSG00000198793 | NFIL3 Facilitates Neutrophil Autophagy, Neutrophil Extracellular Trap Formation and Inflammation During Gout via REDD1-Dependent mTOR Inactivation |
| MYD88 | 4615 | MYD88 innate immune signal transduction adaptor | ENSG00000172936 | Neutrophil Extracellular Traps Promote Inflammatory Responses in Psoriasis via Activating Epidermal TLR4/IL-36R Crosstalk |
| NADPH | 1666 | 2,4-dienoyl-CoA reductase 1 | ENSG00000104325 | SK3 channel and mitochondrial ROS mediate NADPH oxidase-independent NETosis induced by calcium influx |
| NFE2L2 | 4780 | NFE2 like bZIP transcription factor 2 | ENSG00000116044 | Zingerone Inhibits the Neutrophil Extracellular Trap Formation and Protects against Sepsis via Nrf2-Mediated ROS Inhibition |
| NFIL3 | 4783 | Nuclear factor, interleukin 3 regulated | ENSG00000165030 | NFIL3 Facilitates Neutrophil Autophagy, Neutrophil Extracellular Trap Formation and Inflammation During Gout via REDD1-Dependent mTOR Inactivation |
| NFKB1 | 4790 | Nuclear factor kappa B subunit 1 | ENSG00000109320 | Neutrophil Extracellular Traps (NETs) Promote Non- Small Cell Lung Cancer Metastasis by Suppressing lncRNA MIR503HG to Activate the NF-κB/NLRP3 Inflammasome Pathway |
| NLRP3 | 114548 | NLR family pyrin domain containing 3 | ENSG00000162711 | NLRP3 Inflammasome Assembly in Neutrophils Is Supported by PAD4 and Promotes NETosis Under Sterile Conditions |
| NLRP3 | 114548 | NLR family pyrin domain containing 3 | ENSG00000162711 | Neutrophil Extracellular Traps (NETs) Promote Non- Small Cell Lung Cancer Metastasis by Suppressing lncRNA MIR503HG to Activate the NF-κB/NLRP3 Inflammasome Pathway |
| NOX4 | 50507 | NADPH oxidase 4 | ENSG00000086991 | The atypical small GTPase GEM/Kir is a negative regulator of the NADPH oxidase and NETs production through macroautophagy |
| ORAI1 | 84876 | ORAI calcium release-activated calcium modulator 1 | ENSG00000276045 | Excessive neutrophil extracellular trap formation induced by Porphyromonas gingivalis lipopolysaccharide exacerbates inflammatory responses in high glucose microenvironment |
| P2RX1 | 5023 | Purinergic receptor P2X 1 | ENSG00000108405 | Targeting P2RX1 alleviates renal ischemia/reperfusion injury by preserving mitochondrial dynamics |
| PADI4 | 23569 | Peptidyl arginine deiminase 4 | ENSG00000159339 | PAD4 Deficiency Improves Bleomycin-induced Neutrophil Extracellular Traps and Fibrosis in Mouse Lung |
| PARVB | 29780 | Parvin beta | ENSG00000188677 | DNA of neutrophil extracellular traps promotes cancer metastasis via CCDC25 |
| PF4 | 5196 | Chemokine (C-X-C motif) ligand 4 | ENSG00000163737 | Insights in ChAdOx1 nCoV-19 vaccine-induced immune thrombotic thrombocytopenia |
| PIK3CA | 5290 | Phosphatidylinositol-4,5-bisphosphate 3- kinase | ENSG00000121879 | Phosphatidylinositol-3-kinase α catalytic subunit gene somatic mutations in bronchopulmonary neuroendocrine tumours |
| PIK3CG | 5294 | phosphatidylinositol- 4,5-bisphosphate 3- kinase catalytic subunit gamma | [ENSG00000105851](http://www.ensembl.org/id/ENSG00000105851) | Immune mechanism of low bone mineral density caused by ankylosing spondylitis based on bioinformatics and machine learning |
| PINK1 | 65018 | PTEN induced kinase 1 | ENSG00000158828 | The three ‘P’s of mitophagy: PARKIN, PINK1, and  post-translational modifications |
| PKM | 5315 | Pyruvate kinase M1/2 | ENSG00000067225 | Phosphatidylinositol-3-kinase α catalytic subunit gene somatic mutations in bronchopulmonary neuroendocrine tumours |
| PPARGC1A | 10891 | PPARG coactivator 1 alpha | ENSG00000109819 | Neutrophil Extracellular Traps Drive Mitochondrial Homeostasis in Tumors to Augment Growth |
| PROCR | 10544 | Protein C receptor | ENSG00000101000 | Endothelial cell protein C receptor regulates neutrophil extracellular trap-mediated rheumatoid arthritis disease progression |
| PRTN3 | 5657 | Proteinase 3 | ENSG00000196415 | Cathepsin C promotes breast cancer lung metastasis by modulating neutrophil infiltration and neutrophil extracellular trap formation |
| PRTN3 | 5657 | Proteinase 3 | ENSG00000196415 | Cathepsin C promotes breast cancer lung metastasis by modulating neutrophil infiltration and neutrophil extracellular trap formation |
| PTGER2 | 5732 | prostaglandin E receptor 2 | ENSG00000125384 | Immune mechanism of low bone mineral density caused by ankylosing spondylitis based on bioinformatics and machine learning |
| RIPK1 | 8737 | Receptor interacting serine/threonine kinase 1 | ENSG00000137275 | A Role for Receptor-Interacting Protein Kinase-1 in Neutrophil Extracellular Trap Formation in Patients with Systemic Lupus Erythematosus: a Preliminary Study |
| RIPK3 | 11035 | Receptor interacting serine/threonine kinase 3 | ENSG00000129465 | The pseudokinase MLKL activates PAD4-dependent NET formation in necroptotic neutrophils |
| S100A12 | 6283 | S100 calcium binding protein 2 | ENSG00000163221 | Two novel enzyme-linked immunosorbent assays (ELISAs), designed to detect complexes containing DNA, leucocyte calprotectin and S100A12 proteins, were generated for improved specificity and rapid measurement of neutrophil extracellular traps (NETs) |
| S100A8 | 6279 | S100 calcium binding protein A8 | ENSG00000143546 | S100A8/A9 Is a Marker for the Release of Neutrophil  Extracellular Traps and Induces Neutrophil Activation |
| S100A9 | 6280 | S100 calcium binding protein A9 | ENSG00000163220 | S100A8/A9 Is a Marker for the Release of Neutrophil Extracellular Traps and Induces Neutrophil Activation |
| S1PR2 | 9294 | Sphingosine-1-phosphate receptor 2 | ENSG00000267534 | Inhibition of Sphingosine-1-Phosphate Receptor 2 Prevents Thoracic Aortic Dissection and Rupture |
| SELP | 6403 | P-selectin | ENSG00000174175 | P-selectin promotes neutrophil extracellular trap formation in mice |
| SGK1 | 6446 | Serum/glucocorticoid regulated kinase 1 | ENSG00000118515 | Hepatocyte SGK1 activated by hepatic ischemia-reperfusion promotes the recurrence of liver metastasis via IL-6/STAT3 |
| SGK1 | 6446 | Serum/glucocorticoid regulated kinase 1 | ENSG00000118515 | Hepatocyte SGK1 activated by hepatic ischemia- reperfusion promotes the recurrence of liver metastasis via IL-6/STAT3 |
| SIGLEC14 | 10049587 | Sialic acid binding Ig like lectin 4 | ENSG00000254415 | Sialylated Cervical Mucins Inhibit the Activation of Neutrophils to Form Neutrophil Extracellular Traps in Bovine in vitro Model |
| SPP1 | 6696 | Secreted phosphoprotein 1 | ENSG00000118785 | Pan-Cancer Analysis Reveals a Distinct Neutrophil Extracellular Trap-Associated Regulatory Pattern |
| SPP1 | 6696 | Secreted phosphoprotein 1 | ENSG00000178726 | Pan-Cancer Analysis Reveals a Distinct Neutrophil Extracellular Trap-Associated Regulatory Pattern |
| SRC | 6714 | SRC proto-oncogene, non-receptor tyrosine kinase | ENSG00000197122 | Src family kinases and Syk are required for neutrophil extracellular trap formation in response to β-glucan particles |
| STAT3 | 6774 | Signal transducer and activator of transcription 3 | ENSG00000168610 | Neutrophil extracellular traps and their histones promote Th17 cell differentiation directly via TLR2 |
| SUCNR1 | 56670 | Succinate receptor 1 | ENSG00000198829 | Succinic acid exacerbates experimental autoimmune uveitis by stimulating neutrophil extracellular traps formation via SUCNR1 receptor |
| SYK | 6850 | Spleen associated tyrosine kinase | ENSG00000165025 | Src family kinases and Syk are required for neutrophil extracellular trap formation in response to β-glucan particles |
| THBD | 7056 | Thrombomodulin | [ENSG00000178726](http://www.ensembl.org/id/ENSG00000178726) | A Review of Neutrophil Extracellular Traps (NETs) in Disease: Potential Anti-NETs Therapeutics |
| TIMP1 | 7076 | TIMP metallopeptidase inhibitor 1 | ENSG00000102265 | TIMP1 Triggers Neutrophil Extracellular Trap Formation in Pancreatic Cancer |
| TKT | 7086 | Transketolase | ENSG00000163931 | Transketolase and vitamin B1 influence on ROS- dependent neutrophil extracellular traps (NETs) formation |
| TLR2 | 7097 | Toll like receptor 2 | ENSG00000137462 | Neutrophil extracellular traps and their histones promote Th17 cell differentiation directly via TLR2 |
| TLR4 | 7099 | Toll like receptor 4 | ENSG00000136869 | Neutrophil Extracellular Traps Promote Inflammatory Responses in Psoriasis via Activating Epidermal TLR4/IL-36R Crosstalk |
| TLR4 | 7099 | Toll-like receptor 4 | ENSG00000136869 | Neutrophil Extracellular Traps Promote Inflammatory Responses in Psoriasis via Activating Epidermal TLR4/IL-36R Crosstalk |
| TLR7 | 51284 | Toll like receptor 7 | ENSG00000196664 | Partners in crime: Autoantibodies complicit in COVID-19 pathogenesis |
| TLR8 | 51311 | Toll like receptor 8 | ENSG00000101916 | Neutrophil extracellular traps mediate a host defense response to human immunodeficiency virus-1 |
| TLR9 | 54106 | Toll like receptor 9 | ENSG00000239732 | Glycyrrhizin alleviates sepsis-induced acute respiratory distress syndrome via suppressing of HMGB1/TLR9 pathways and neutrophils extracellular traps formation |
| TNF | 7124 | Tumor necrosis factor-alpha | ENSG00000232810 | Immune mechanism of low bone mineral density caused by ankylosing spondylitis based on bioinformatics and machine learning |
| TNFAIP3 | 7128 | TNF alpha induced protein 3 | ENSG00000118503 | Genetic variations in A20 DUB domain provide a genetic link to citrullination and neutrophil extracellular traps in systemic lupus erythematosus |
| WASL | 8976 | WASP like actin nucleation promoting factor | ENSG00000106299 | Host liver-derived extracellular vesicles deliver miR- 142a-3p induces neutrophil extracellular traps via targeting WASL to block the development of Schistosoma japonicum |
| AGER | 177 | Advanced glycosylation end- product specific receptor | ENSG00000204305 | Neutrophil extracellular traps mediate the crosstalk between glioma progression and the tumor microenvironment via the HMGB1/RAGE/IL-8 axis |
| Ros1 | 6098 | MucR family transcriptional regulator Ros | ENSG00000047936 | Exogenous hydrogen sulfide inhibits neutrophils extracellular traps formation via the HMGB1/TLR4/p-38 MAPK/ROS axis in hyperhomocysteinemia rats |
| [EPCAM](https://www.ncbi.nlm.nih.gov/gene/4072) | 4072 | Epithelial cell adhesion molecule | ENSG00000119888 | Neutrophil extracellular traps drive epithelial-mesenchymal transition of human colon cancer |
| CDH1 | 999 | Cadherin 1 | ENSG00000039068 | Neutrophil extracellular traps drive epithelial- mesenchymal transition of human colon cancer |
| ZEB1 | 6935 | Zinc finger E-box binding homeobox 1 | ENSG00000148516 | Neutrophil extracellular traps drive epithelial- mesenchymal transition of human colon cancer |
| VIM | 7431 | Vimentin | ENSG00000026025 | Neutrophil extracellular traps induced by VP1 contribute to pulmonary edema during EV71 infection |
| ENTPD4 | 9583 | Ectonucleoside triphosphate diphosphohydrolase 4 | ENSG00000197217 | Neutrophil extracellular traps in acute coronary syndrome |
| FN1 | 2335 | Fibronectin 1 | ENSG00000115414 | Integrin-dependent cell adhesion to neutrophil extracellular traps through engagement of fibronectin in neutrophil-like cells |
